# Anti-Atherogenic Effect of 10% Supplementation of Anchovy (*Engraulis encrasicolus*) Waste Protein Hydrolysates in ApoE-Deficient Mice

**DOI:** 10.3390/nu13072137

**Published:** 2021-06-22

**Authors:** Jessica Maria Abbate, Francesco Macrì, Francesca Arfuso, Carmelo Iaria, Fabiano Capparucci, Carmelo Anfuso, Antonio Ieni, Luca Cicero, Giovanni Briguglio, Giovanni Lanteri

**Affiliations:** 1 Department of Veterinary Sciences, University of Messina, Polo Universitario Annunziata, 98168 Messina, Italy; jabbate@unime.it (J.M.A.); francesco.macri@unime.it (F.M.); francesca.arfuso@unime.it (F.A.); 2 Department of Chemical, Biological, Pharmaceutical and Environmental Sciences, University of Messina, Polo Universitario Papardo, 98166 Messina, Italy; fabiano.capparucci@unime.it (F.C.); giovanni.lanteri@unime.it (G.L.); 3 IRCCS Centro Neurolesi Bonino Pulejo, 98124 Messina, Italy; carmelo.anfuso@irccsme.it; 4 Department of Human Pathology of Adult and Evolutive Age “Gaetano Barresi”, Section of Pathology, University of Messina, 98125 Messina, Italy; antonio.ieni@unime.it; 5 Zooprophylactic Institute of Sicily “A. Mirri” (IZS), 90129 Palermo, Italy; luca.cicero@izssicilia.it; 6 Veterinary Practitioner, 98168 Messina, Italy; giobri9@gmail.com

**Keywords:** atherosclerosis, ApoE-knockout mice, fish proteins, fish protein hydrolysates, anchovy by-products

## Abstract

Fish protein consumption exerts beneficial metabolic effects on human health, also correlating with a decreased risk for cardiovascular disease. Fish waste contains high amount of proteins and utilization may offer the opportunity for generating compounds advantageous for human health. Especially, fish waste protein hydrolysates beneficially influence pathways involved in body composition, exerting anti-inflammatory and antioxidant activities, making their potential supplementation in human disorders of increased interest. This study assessed the effect of a 10% (*w/w*) anchovy waste protein hydrolysate (APH) diet for 12 weeks in reducing atherosclerosis in ApoE^−/−^ mice, through histological and immunohistochemical methods. In addition, monitoring of plaque development was performed, using high-frequency ultrasound and magnetic resonance imaging. Overall, the APH diet attenuated atherosclerotic plaque development, producing a regression of arterial lesions over time (*p* < 0.05). Twelve weeks on an APH diet had an anti-obesity effect, improving lipid metabolism and reducing hepatic enzyme activity. A significant reduction in plaque size and lipid content was observed in the aortic sinus of APH-fed mice, compared to the control (*p* < 0.001), whereas no differences in the extracellular matrix and macrophage recruitment were observed. Supplementation of APH significantly attenuates atherosclerosis in ApoE^−/−^ mice, exerting a lipid-lowering activity. The opportunity to use fish waste protein hydrolysates as a nutraceutical in atherosclerosis is worthy of future investigations, representing a low cost, sustainable, and nutritional strategy with minimal environmental impact.

## 1. Introduction

Globally, atherosclerotic cardiovascular disease is a primary cause of mortality, accounting for at least 30% of all deaths annually in most developed and developing countries [1]. Atherosclerosis is a complex inflammatory disease of the large- and medium-sized arteries, characterized by gradual lipid accumulation within the intima, further promoting extracellular matrix synthesis and remodelling, and chronic inflammation, involving cells of both the innate and acquired immune system [2,3]. Gradually, thickening of the arterial wall limits the blood flow, and disease often remains asymptomatic for years, with sudden death as the first clinical manifestation [1].

Several risk factors for atherosclerotic disease have been recognized in humans, including adiposity, cholesterol blood levels, hypertension, diabetes mellitus, vascular endothelial dysfunctions, and chronic inflammatory conditions [1,4,5]. In particular, dyslipidaemia and oxidative stress play a central role in the pathogenesis of atherosclerosis [6]. Alterations in lipoprotein plasma levels, especially for low-density lipoprotein (LDL) cholesterol, and concurrent vascular endothelial dysfunction and damage, are responsible for the accumulation of lipoproteins in the subendothelial space of the arterial intima and subsequent recruitment of circulating monocytes [6]. Additionally, the chronic vascular oxidative stress that characterizes dyslipidaemic states and other dysmetabolic disorders is responsible for lipoprotein oxidation, and oxidized LDLs (ox-LDLs) stimulate chronic inflammation, further promoting vascular smooth muscle cell proliferation and extracellular matrix remodeling [2]. Monocytes recruited in the arterial intima convert into macrophages and act as ox-LDL scavengers, becoming lipid laden and converting into foam cells [7]. Although useful in removing ox-LDLs, the prolonged retention of ox-LDLs in macrophages is afterwards responsible for their cell death, which contributes to aggravate pre-existing vascular lesions [7]. 

Since dietary habits and lifestyle play a chief role in the prevention and treatment of atherosclerotic cardiovascular disease in humans, the search for effective nutritional strategies in reducing risk for disease is worthy of investigation [4]. In particular, the crucial role of dyslipidaemia, oxidative stress, and inflammation in the pathogenesis and severity of the disease has spurred the search for nutrients and food-derived bioactive compounds, exerting antioxidant and anti-inflammatory activities, also improving lipid metabolism [8,9,10,11]. Fish protein is one of the most consumed dietary protein sources and the correlation between fish protein consumption and decreasing risk for cardiovascular disease has been documented [12,13]. The fish processing industry generates more than 60% of the fish biomass as waste, characterized by a certain amount of high-quality proteins, still poorly used and exploited [14,15,16]. Several biological and chemical technologies have been investigated to generate protein-rich compounds from fish by-products [8,16,17,18], and currently, enzymatic hydrolysis is considered the most efficient method that allows added-value protein hydrolysates to be generated, and it has proven to exert several biological and nutraceutical activities [8,16,17,18]. Noteworthy, protein hydrolysates from fish waste beneficially influence the pathways involved in body composition, and lipid and glucose metabolism, and also possess desirable anti-inflammatory, immunomodulatory, and antioxidant activities [9,19,20,21]. In consideration of the high-quality protein content and amino acid profiles, protein hydrolysates from fish have attracted great interest in recent years, and promising results obtained encourage their supplementation in current therapeutic strategies in numerous chronic diseases in humans [16,17,18,20,22,23]. 

The anchovy (*Engraulis encrasicolus*) is a common fish species of the Mediterranean Sea, known to produce high-value bioactive peptides under enzymatic hydrolysis, with nutraceutical potential only partially explored to date [24]. A recent survey carried out by our research group demonstrated that 12-week administration of 10% (*w/w*) anchovy protein hydrolysates (APH) in a high-fat diet produced a significant anti-obesity effect, improving lipid metabolism and reducing hepatic steatosis and liver damage in ApoE^−/−^ mice [25]. Additionally, APH significantly produced anti-inflammatory and antioxidant effects, downregulating the expression of pro-inflammatory cytokines and modulating the expression of oxidative stress-related genes in the aorta and heart tissue in the same ApoE^−/−^ mice [26]. Therefore, in view of the promising results previously obtained [26], the purpose of the current study was to assess the influence of anchovy viscera protein hydrolysates in reducing atherosclerosis in ApoE^−/−^ mice used in previous studies through histological and immunohistochemical methods. Additionally, imaging studies (i.e., high-frequency ultrasonography and magnetic resonance imaging) were performed to monitor atherosclerotic plaque development during the experimental trial.

## 2. Materials and Methods

### 2.1. Animals and Experimental Diet

This study was approved by the Animal Welfare Organization (OPBA) (approval no. 771/2018-PR; 4 October 2018). In vivo experimental procedures were conducted in accordance with national (D. Lgs 2014/26) and European regulations (EU Directive 2010/63). In vivo methods were performed at the Experimental Zooprophylactic Institute of Sicily “A. Mirri”, Palermo (cod 28875, Ministerial authorization 14/2015-UT). 

Six-month-old female B6.129P2-ApoE^−/−^ mice (C57BL/6 genetic background), enrolled in previous studies carried out by of our research group [25,26], were used in this experimental trial. Mice were allocated to sterile filter-top cages and kept on a 12-h light/dark cycle, in standard laboratory conditions (21 ± 1 °C, 55 ± 5% humidity). After 1-week acclimatization, mice were randomly allocated into two groups (*n* = 6/group) and individually weighed. For 12 weeks, mice included in both groups were fed a high-fat diet (HFD) (TD.88137 Envigo; Indianapolis, United States) to enhance hypercholesterolemia and exacerbate atherosclerosis. For mice included in the experimental group, the commercial diet was modified and 10% (*w/w*) of casein was replaced with the same amount of anchovy protein hydrolysates (*w/w*) (APH). Briefly, protein hydrolysates were prepared by enzymatic hydrolysis of anchovy waste (viscera) using a combination of commercial proteases (Promatex, Flavourzyme 500 MG, Alcalase 2.4 L; 1.1,1.1,0.9, *w/w*; Novozymes China Inc.; Guangzhou, China), and the amino acid composition was further determined by means of high-performance liquid chromatography (HPLC) (Table 1), as described by Mangano et al. (2019). Mice of both groups received water and chow ad libitum during acclimatization and the experimental trial.

### 2.2. Experimental Procedures

During the study, mice were checked daily for health status, monitored for food intake, and weighed weekly. After 12 weeks (Study day 84; T84), the mice were euthanized with an overdose of anaesthetic (isoflurane > 5%). Blood was sampled, stored in a 2.5-mL cloth activator tube and centrifuged to obtain serum. Total cholesterol (TC; mg/dL), triglycerides (TG; mg/dL), aspartate amino transferase (AST; UI/L), and alanine aminotransferase (ALT; UI/L) serum concentration were determined using an automated clinical chemistry analyzer (Konelab 60I; Thermo Electron Corporation, Vantaa, Finland) and commercially available kits (Thermofisher, Fisher Diagnostics, Thermo Fisher Scientific Inc., Middletown, OH, USA). 

Necropsy was performed for all mice and hearts were sampled, snap-frozen, stored at −80 °C, and fixed in 10% (*v/v*) neutral buffered formalin solution for further investigations. 

### 2.3. Abdominal High-Frequency Ultrasound 

Abdominal high-frequency ultrasound (hfUS) examination was performed after 8 (Study day 56; T56) and 12 (T84) weeks, to monitor plaque development in the abdominal aorta of all mice, using a high-resolution imaging system (Vevo 2100, FUJIFILM VisualSonics Inc.). Briefly, mice were anesthetized with a 2% isoflurane-oxygen mixture in an isoflurane induction chamber, positioned in the right lateral recumbency on a clinical examination table and placed on a heating platform to reduce procedural stress and prevent hypothermia. After trichotomy, the abdomen was covered with acoustic coupling gel and B-mode of the abdominal aorta was obtained with a higher frequency (22 MHz) probe (MS550, FUJIFILM VisualSonics Inc.). Longitudinal and transverse sections of the abdominal aorta were obtained, placing the probe on the left dorsal plane and the ultrasound beam as perpendicular as possible (orthogonal) to the long axis of the vessels. Plaque area (%) was measured using ImageJ Software on three representative acquired images for each animal, and expressed as the mean value ± SD on *n* = 6 mice per group. 

### 2.4. Magnetic Resonance Imaging 

Magnetic resonance imaging (MRI) was performed immediately after hfUS examination, using a 7T horizontal bore PharmaScan 70/16 US scanner (Bruker, Ettlingen, Germany), equipped with a 23-mm transmit/receive volumetric coil. Axal images were acquired using a slice thickness of 0.7 mm. The field of view used was 20 × 20 mm^2^, resulting in an in-plane resolution of 256 × 256 μm^2^. During MRI, mice were monitored for respiration, and a respiration triggered gradient echo (TE: 35.0 ms), and a T1 Turbo RARE sequence was selected for T1 imaging. A respiratory and an ECG sensor were connected to a monitoring system (SA Instruments, Stony Brook, NY, USA). A respiratory system was placed on the abdomen to monitor the rate and depth of respiration, to reduce motion artefacts during imaging. The flow of anaesthetic gas was constantly regulated to maintain a breathing rate of 50 ± 20 bpm; heart rate was maintained at ~500 bpm with 0.8–1.8% isoflurane and body temperature at 35.0 ± 0.8 °C by blowing hot air into the magnet through a feedback control system. Plaque area (%) was measured on three representative acquired images for each animal and performed blinded, using ImageJ Software and expressed as mean ± SD on *n* = 6 mice per group.

### 2.5. Histopathology 

Formalin-fixed, paraffin-embedded, serial sections (5 μm) of the heart were stained with hematoxylin and eosin (H&E) to assess atherosclerotic plaque composition. Morphometric parameters (i.e., plaque area; plaque/lumen ratio) were determined and expressed as the mean value of three sections of the aortic sinus showing the three cusps of the aortic valves from *n* = 6 mice per group. Snap-frozen, serial cryosections (7 μm) of the aortic sinus were stained with Oil Red O (04-2209-23; Sigma–Aldrich, St. Louis, MO, USA) and Mallory’s Trichrome stainings (04-020802; Bioptica, Milano, Italy) to detect intraplaque lipids and extracellular matrix deposition, respectively. Histological sections were visualized using a Leica DM6B microscope (Leica Camera, Wetzlar, Germany) using Leica Application Suite X software, and images were acquired using a Leica DFC 7000 T. Image J Software was used for quantification of intraplaque lipids and extracellular matrix deposition using images at high magnification (400×), and expressed as the percentage of the positively stained area/total plaque area. Quantification was assessed on three representative sections from *n* = 6 animals per group and performed by a pathologist blinded to the experimental protocol. 

### 2.6. Immunohistochemistry 

Immunohistochemistry was performed on 5 μm paraffin-embedded serial sections of the aortic sinus, using the following antibodies: rat monoclonal anti-F4/80 (Abcam, Cambridge, UK; Product Code: ab16911), rat monoclonal anti-CD3 (Abcam, Cambridge, UK; Product Code: ab16911); mouse monoclonal anti-BDNF (Abcam, Cambridge, UK; Product Code: ab205067); rabbit monoclonal anti-TrkB (Abcam, Cambridge, UK; Product Code: ab187041); rabbit polyclonal anti-FNDC5 (Abcam, Cambridge, UK; Product Code: ab131390). All primary antibodies were incubated at 4 °C overnight. Binding sites were revealed using secondary antibodies, developed from an Avidin-biotin complex (BioSpa 20143, Milano, Italy), and 3,3′-Diaminobenzidine was used as chromogen (DAB; Vector Laboratories, Inc. U.S. Headquarters, Burlingame, CA, USA). Sections were counterstained with Mayer’s haematoxylin (Bio-Optica, Milano, Italy). Positive and negative controls were always included as described in Iaria et al. 2019 [27]. Negative controls were performed omitting the specific antisera and replacing PBS for the primary antibody. 

The Image J Software was used for quantification of positively labelled area for anti- F4/80 and anti-CD3 antibodies and reported as % of positive stained area/total plaque area. Quantification was assessed on three representative sections from *n* = 6 animals per group, and performed blinded. Anti-brain-derived neurotrophic factor (BDNF), tyrosinase receptor B (TrkB), and fibronectin domain-containing protein 5 (FNDC5) expression was assessed qualitatively, expressed as negative or positive labelling, using an optical microscopy. For positively labelled sections, the following parameters were used: diffused (labelling all over the microscopic field), multifocal (labelling restricted to well-defined areas), and focal (labelling of a single well-defined area). 

### 2.7. Statistical Analysis 

Data were presented as mean ± standard deviation (±SD) for *n* = 6 mice per group. All data were tested by Kolmogorov–Smirnov test and were normally distributed (*p* > 0.05). An unpaired Student’s *t*-test was performed to assess statistically significant differences of histological, immunohistochemical, HfUS, and MRI findings between groups (control group: ApoE^−/−^ HFD; experimental group: ApoE^−/−^ HFD+APH). Statistical analysis was performed using GraphPad Prism version 7.00 (GraphPad Software, San Diego, CA, USA).

## 3. Results

### 3.1. Animals and Serum Analysis (Data Previously Published, Abbate et al. 2020)

Data on body weight gain and serum parameters recorded in ApoE^−/−^ HFD and ApoE^−/−^ HFD+APH mice have already been published in a previous study carried out by our research group [25]. However, in the authors’ opinion it is necessary to include the trend of these parameters again, which may be useful for better understanding the findings on atherosclerotic plaque development achieved here. At the inclusion day (study day 0; T0), mice of both groups were weighed (Apo^−/−^ HFD: mean 15.83 ± 0.47 g, range 15.00–16.30 g; Apo^−/−^ HFD + APH: mean 16.00 ± 0.22 g, range 15.70–16.30 g) and body size was determined (Apo^−/−^ HFD and Apo^−/−^ HFD + APH: mean 15.10 ± 0.40 cm). After 12 weeks, body weight gain was lower in APH-fed mice (mean: 22.55 ± 0.43 g, range 22.00–23.20 g), compared to the control group (mean: 23.92 ± 0.19 g, range 23.70–24.20 g) (body size: Apo^−/−^HFD and Apo^−/−^ HFD + APH: mean 16.80 ± 0.60 cm). A statistically significant effect of different diet on body weight gain was recorded throughout the study in mice of both groups. An increasing trend in the body weight gain was observed in mice of both groups (*p* < 0.05), with a percentage increase of 50.47% recorded in ApoE^−/−^ mice fed HFD and 40.94% in ApoE^−/−^ fed HFD + APH. Body weight gain was significantly lower in APH-fed mice compared to the control group, from study day 14 to end of study (*p* < 0.001) (Figure S1). Food intake varied from 3.16 to 6.04 g/day for individual mice throughout the study, with no significant differences between groups related to diet. 

Total cholesterol (TC) and triglyceride (TG) serum concentrations were significantly lower in APH-fed mice (TC: 737.83 ± 64.74; TG: 74.33 ± 5.57 mg/dL) compared to concentrations from HFD-fed mice (TC: 1534.33 ± 0.21; TG: 98.67 ± 9.67 mg/dL) (*p* < 0.001) at 12 weeks. In addition, a significant increase in serum ALT and AST concentrations was observed in HFD-fed mice (ALT: 282.33 ± 15.88; AST: 2889.00 ± 0.16 U/L) compared to APH-fed mice (ALT: 28.33 ± 3.88; AST: 481.17 ± 33.59 U/L).

### 3.2. HFUS and MRI Monitoring

**Figure 1:**
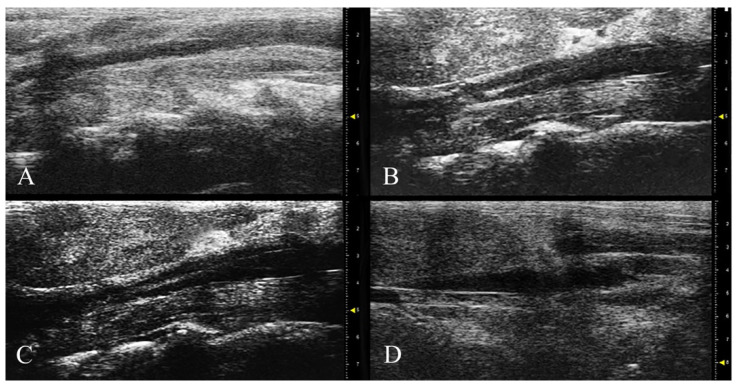
B-mode imaging of the abdominal aorta in longitudinal view. Atherosclerotic plaque (echolucent) protruding into the lumen of the aorta in ApoE^−/−^ HFD+APH at 8 (**A**) and 12 weeks (**B**). Atherosclerotic plaque into the lumen of the abdominal aorta in ApoE^−/−^ HFD at 8 (**C**) and 12 weeks (**D**).

B-mode of the abdominal aorta in longitudinal plane showed a tubular structure with well-defined thin smooth, hyperechoic outer walls. The abdominal aorta appeared circular to oval in cross-sections. The lumen was anechoic lumen because of the echo-free blood inside. The anechoic content of the aorta was interrupted by the presence of atheromatous echogenic plaques of different sizes. In particular, the area (%) occupied by atherosclerotic plaque differed between groups, with a statistically significant lower area (*p* < 0.05) recorded in APH-fed mice compared to the control group in both follow-ups (i.e., 8, 12 weeks) (T56: 39.65 ± 3.23% vs. 50.39 ± 0.98%) (T84: 28.41 ± 3.12% vs. 59.86 ± 5.10%) (Figure 1). Moreover, the regression of the plaque area in APH-fed mice throughout the monitoring period resulted in statistically significant with lower values at 12 weeks compared to 8 weeks (*p* < 0.05). 

**Figure 2:**
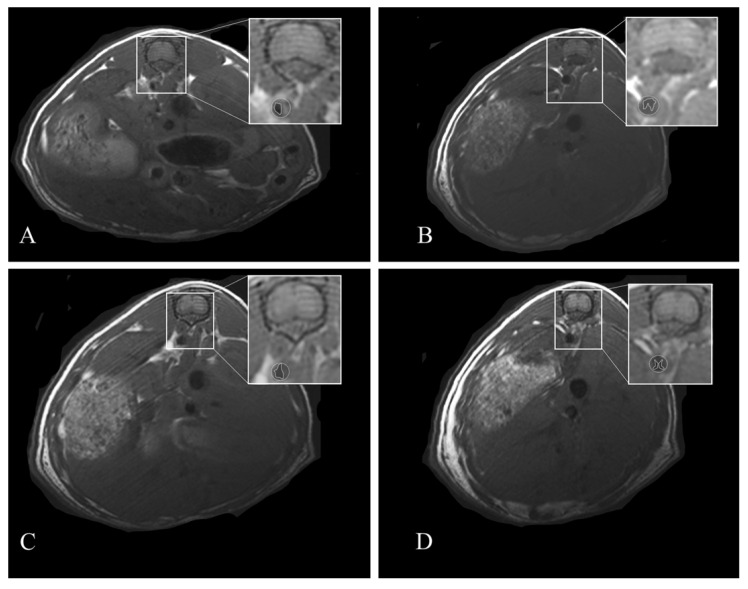
In vivo magnetic resonance cross sectional images (T1 rare) of abdominal aorta. Atherosclerotic plaque protruding into the lumen of the aorta in ApoE^−/−^ HFD+APH at 8 (**A**) and 12 weeks (**B**). Atherosclerotic plaque in the abdominal aorta in ApoE^−/−^ HFD at 8 (**C**) and 12 weeks (**D**).

Minimal respiratory motion artefacts were observed on MR images of the abdomen. The abdominal aorta was visualized by MR in all mice. The vascular lumen appeared dark in the MR images because of the blood-flow-related signal loss from the spin-echo image acquisition. The MR images in mice abdominal aorta in both groups showed progression of an atherosclerotic plaque. The atheromatous progression appears slower in the experimental group than in the control group. MRI showed a statistically significant lower plaque area in APH-fed mice (*p* < 0.05) at 8 weeks (44.80 ± 2.97%) compared to the control HFD-fed mice (65.90 ± 5.12%) as well as at 12 weeks (ApoE^−/−^ HFD + APH: 40.40 ± 1.19%; ApoE^−/−^ HFD: 69.50 ± 4.37%) (Figure 2). 

### 3.3. Atherosclerotic Plaque Development 

**Figure 3:**
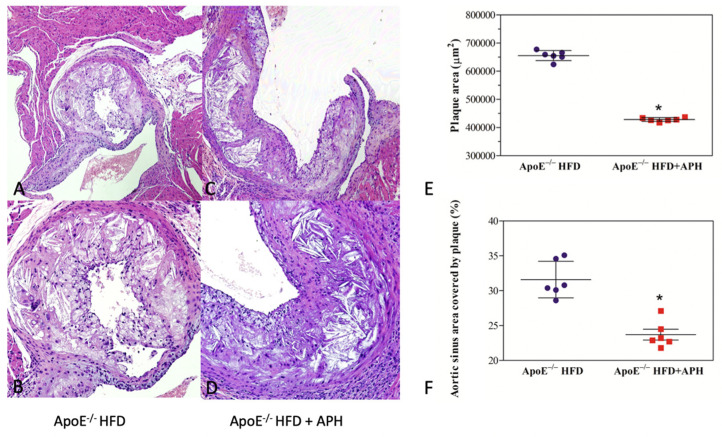
Histological characterization of atherosclerotic plaques in aortic sinus of ApoE^−/−^ HFD (control) (**A**,**B**) and ApoE^−/−^ HFD +APH mice (**C**,**D**) (HE). Quantification of plaque area, with a significant reduction in plaque area in ApoE^−/−^ HFD+APH compared to the ApoE^−/−^ HFD control mice (**E**) (*p* < 0.001). Significant reduction in percentage of aortic sinus covered by plaque in ApoE^−/−^ HFD + APH compared to the ApoE^−/−^ HFD mice (*p* < 0.001) (**F**). Quantification is expressed as mean ± SD for *n* = 6 animals per group. (Magnification 10×, (**A**,**C**)); (magnification 20×, (**B**,**D**).

Data analysis showed a statistically significant effect of diet on the values of plaque area and percentages of aortic sinus area covered by plaques (*p* < 0.001). A significant reduction in plaque areas was observed in APH-fed mice (428,492.50 ± 6,806.60 um^2^) compared to the HFD-control mice (655,582.15 ± 17,908.94 um^2^), corresponding to 25.00 ± 1.02% vs. 34.00 ± 2.54% of the aortic sinus surface covered by plaques (Figure 3).

**Figure 4:**
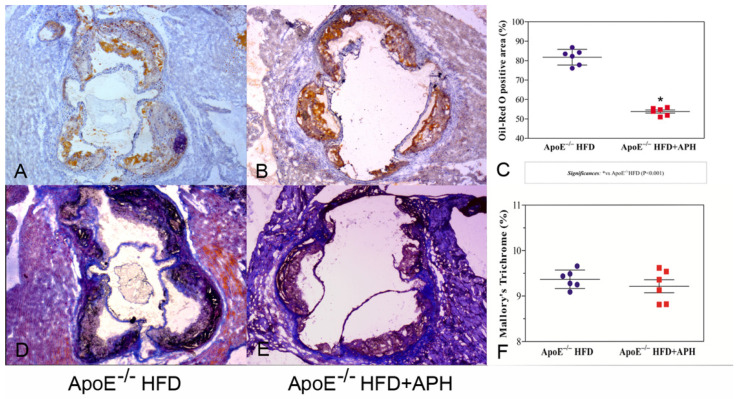
Histochemical characterization of atherosclerotic plaques in aortic sinus of ApoE^−/−^ HFD + APH and ApoE^−/−^ HFD (control) mice. Representative photomicrographs and quantification of lipid deposition in plaque area (**A**–**C**). Representative photomicrographs and quantification of extracellular matrix deposition (**D**–**F**). Quantification of lipids and extracellular matrix deposition was expressed as percentage of positively stained area/total plaque area. Quantification is presented as mean ± SD for n = 6 animals per group. (Magnification 5×).

Histochemical characterization of atherosclerotic plaques showed differences in plaque composition between groups. In detail, a significant difference in lipid composition was observed in Oil Red O sections, with a lower percentage of positively stained area recorded in APH-fed mice (53.84 ± 1.97%) compared to HFD-fed control mice (81.79 ± 4.05%) (*p* < 0.001), demonstrating a lipid-lowering activity of APH on atherosclerotic plaques (Figure 4).

**Figure 5:**
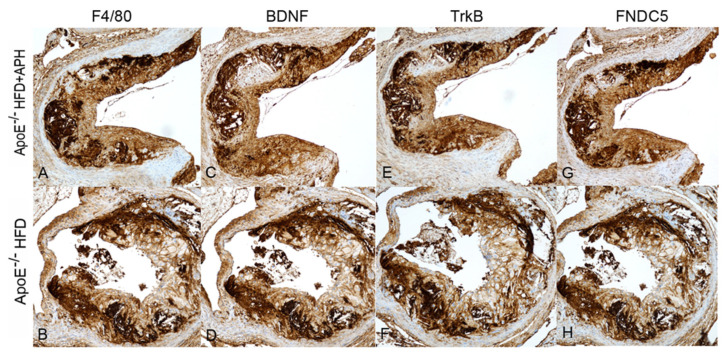
Immunohistochemical characterization of atherosclerotic plaques in aortic sinus of ApoE^−/−^ HFD+APH and ApoE^−/−^ HFD (control) mice. Representative photomicrographs of F4/80 expression (**A**,**B**). Representative photomicrographs of BDNF (**C**,**D**), TrkB (**E**,**F**), and FNDC5 (**G**,**H**) expression in atherosclerotic plaques (magnification 10×).

Conversely, no significant differences in extracellular matrix (ApoE^−/−^ HFD + APH: 9.21 ± 0.70% vs. ApoE^−/−^ HFD: 9.37 ± 0.20%) (Figure 4) and macrophages (ApoE^−/−^ HFD: 32.33 ± 0.64% vs. ApoE^−/−^ HFD + APH: 32.00 ± 0.80%) (Figure 5) were observed between groups, in Mallory’s Trichrome and F4/80-labeled sections. Lymphocytes were not detected in plaques from either group as no CD3 expression was observed, whereas plaque expression of BDNF, TrkB, and FNDC5 was diffused in aortic sinus plaques of mice of both groups, and therefore no differences in expression levels were observed (Figure 5). 

## 4. Discussion

The beneficial effect of fish consumption in decreasing risk for cardiovascular disease has been highlighted by observational studies through a meta-analysis approach [12,13], and, despite beneficial properties being mainly linked to the omega-3 polyunsaturated fatty acid content, emerging evidence from studies suggests that fish proteins also exert essential metabolic effects enhancing lipid and glucose metabolism and ameliorating body composition [8,9,10,16,18]. 

Animals models in which more rapid changes occur are useful for assessing the influence of nutritional strategies and pharmacological interventions in delaying the formation of atherosclerotic plaques [28], and the targeted deletion of the apoE gene in ApoE^−/−^ mice leads to severe hypercholesterolemia and spontaneous atherosclerosis [29].

In this study, the influence of 10% (*w/w*) anchovy protein hydrolysate supplementation for 12 weeks in attenuating atherosclerotic plaque formation was assessed in ApoE^−/−^ mice, as an atherosclerosis-prone mouse model. High-frequency ultrasound and MRI were performed in monitoring atherosclerotic plaque development in the abdominal aorta, whereas histochemistry and immunohistochemistry analyses were carried out for quantification of atherosclerosis in the aortic sinus. Overall, 12 weeks on an APH diet significantly produced a lower body weight gain, and a significant reduction in total cholesterol and triglycerides, as well as liver enzyme serum concentration. Noteworthy, imaging studies showed a significant reduction in plaque development in APH-fed mice compared to the control group at 8 and 12 weeks, and, interestingly, a regression of plaques in APH-fed mice at 12 weeks compared to previous follow-up. In addition, 12 weeks on an APH diet reduced atherosclerotic plaque formation in the aortic sinus, reducing lipid accumulation, compared to mice from the control group.

The apolipoprotein E-knockout mouse is a widely used animal model to study the genetics and pathogenesis of atherosclerosis, since spontaneously develop the morphological changes, as well as the oxidative and molecular modifications that characterize the disease in humans within 6–10 weeks [30]. In the current survey, mice were fed a high-fat diet to enhance hypercholesterolemia and accelerate plaque formation. Additionally, in view of the well-known higher susceptibility to atherosclerosis of females compared to males with the same genetic background [31], only female mice were enrolled. Indeed, it has been well demonstrated that the genes encoded by the sex chromosomes and differences in the metabolism of trimethylamine-N-oxide (TMAO), a risk factor for atherosclerosis [32], produce more voluminous aortic atherosclerotic plaque in females [31]. 

Imaging studies performed are non-invasive diagnostic techniques useful in monitoring the development of atherosclerotic plaques in laboratory animals, and thus provide an important contribution in disease monitoring. Development of the high-frequency ultrasound method has increased the spatial and temporal resolution of images, allowing differences in aortic dimensions to be better appreciated [33]. In addition, MRI has emerged as a diagnostic technique for serial non-invasive monitoring of progression or regression of the atherosclerotic arterial lesions in mouse models [34], and MRI utility in examining plaque regression has been demonstrated in transplant models [35,36]. In accordance with the above, in the current study both high-frequency ultrasound and MRI allowed us to appreciate differences in plaque size between groups over time, with a significant slowdown in plaque development already recorded at 8 weeks in APH-fed mice. Interestingly, a reduction in plaque size was observed in APH-fed mice at 12 weeks compared to the 8-week follow-up. Therefore, data from imaging studies suggest that APH administration in a high-fat diet produced not only an attenuation in plaque development, but more interestingly, APH seemed to produce plaque regression over time. It is possible to hypothesize that the anchovy protein hydrolysates indirectly ameliorated the plaque through the improvement of the lipid profile of ApoE^−/−^ HFD + APH. As a matter of fact, it has been established that aggressive lipid profile modification, especially with cholesterol-lowering therapy, reduces atherosclerotic progression and induces plaque regression in animal models, stabilizing the plaque and reversing the remodelling of the arterial wall [37,38]. Plasma lipoprotein level improvement produced by a lowering LDL-cholesterol diet induces plaque regression characterized by the improvement of reverse cholesterol transport, reduction in foam cells, and phenotypic switch of retained macrophages from pro-inflammatory to anti-inflammatory cells, with the clearance of necrotic debris and tissue reparation [39]. As already shown [25], anchovy protein hydrolysates exert an anti-obesity effect, ameliorating lipid metabolism, especially for cholesterol serum concentration, leading to lower deposition of cholesterol LDLs in the arterial wall, as well as in lower hepatic fat content and hepatocyte injury [25]. Similarly, 5% (*w/w*) supplementation of salmon protein hydrolysates produced a marked hypocholesterolaemic and anti-atherogenic effect in ApoE^−/−^ mice [20]. Of note, the amino acid composition of anchovy protein hydrolysates used in this study showed a high content of essential amino acids, including proline, alanine, leucine, and aromatic acids [24]. In particular, proline may play an important role in cholesterol metabolism also exerting a protective effect against dangerous free radicals, and thus reducing the risk for cardiovascular diseases, including atherosclerosis [26,40].

Twelve weeks on an APH diet significantly attenuated atherosclerotic plaque development in the aortic sinus. Additionally, quantification of Oil Red O stained area revealed a 30% reduction in lipid plaque accumulation in APH-fed mice compared to the control group. On the contrary, although the connective tissue and macrophage-labelled area were slightly greater in control mice compared to APH-fed mice, differences were not significant, demonstrating that APH supplementation did not influence extracellular matrix synthesis and remodelling, nor macrophage recruitment. The plaque composition influenced its stability and vulnerability to rupture, which represented the leading cause of ischemic cardiac and cerebral acute events and strokes in human patients [1]. Instable plaque often contains a thin fibrous cap and abundant inflammatory cells, which secrete several cytokines harmful to plaque stabilization. In particular, matrix metalloproteinases (MMPs), a group of proteolytic enzymes, play an important role in chronic degenerative diseases, including atherosclerosis, involved in vascular remodelling and contributing to endothelial cell integrity and vascular smooth muscle cells migration. Nevertheless, although useful in assessing plaque composition, ApoE^−/−^ mice do not seem to be susceptible to plaque rupture even on a high-fat diet over a year, limiting the employment of this animal model in studying pathophysiological complications that characterize atherosclerosis in humans. 

Anchovy protein hydrolysates did not influence the content of macrophages in the atherosclerotic plaques. It has been established that in the setting of atherosclerotic lesions, macrophages display different phenotypes that influence their own function, promoting lesion progression or regression [41]. In detail, the different phenotypes affect the ability to engulf ox-LDL, tissue remodelling and repair, affecting their survival and the ability to perform efferocytosis [41]. Which phenotype predominates in aortic plaques here analyzed and whether APH may induced the M1 or the M2 phenotype require further investigations. 

Expression of BDNF, TrkB, and FNDC5 was diffuse in aortic sinus plaques from mice of both groups. BDNF is a neurotrophin shown to be expressed in atheromatous intima, adventitia, and endothelial cells of coronary arteries in humans, where it seems to be implicated in the pathogenesis of atherosclerosis, playing a primary role in atherogenesis and in plaque stability [42]. In particular, BDNF promotes angiogenesis and endothelial cell development, through TrkB, which maintains endothelial barrier integrity by promoting VE-cadherin synthesis [43]; endothelial BDNF/TrkB signaling regulates the shedding of VE-cadherin and protects against atherosclerotic lesion development in ApoE^−/−^ mice [43]. Therefore, the BDNF/TrkB pathway plays a protective role in atherosclerosis and pathway deficiency has been proven to accelerated development of atherosclerotic lesions in ApoE^−/−^ mice [43]. FNDC5 is a membrane protein comprising a short cytoplasmic domain and an ectodomain consisting of a fibronectin type III [44,45]. In atherosclerosis, FNDC5 inhibited ox-LDL-induced foam cell formation and monocyte adhesion in vascular smooth muscle cells (VSMCs) [46]. Noteworthy, FNDC5 overexpression prevents high-fat diet-induced hyperlipidaemia, hepatic lipid accumulation, and impaired fatty acid-b-oxidation and autophagy in liver [47], also ameliorating hyperlipidaemia and enhancing lipolysis in the adipose tissue of obese mice [48]. 

## 5. Conclusions

The results obtained in the current study demonstrate that an APH diet significantly slows down the development of atherosclerotic plaques with a significant reduction in lipid accumulation. Additionally, an APH diet seems to produce plaque regression over time. By virtue of an anti-atherogenic effect, anchovy waste protein hydrolysates could be successfully employed as nutraceuticals in atherosclerotic cardiovascular disease prevention and treatment in the future. The possibility of obtaining high-value compounds from fish waste to be employed in chronic human disorders is worthy of future investigation, representing a very low cost, sustainable nutritional strategy with minimal environmental impact, which may be explored in the prevention and treatment of several chronic human metabolic diseases.

## Figures and Tables

**Table 1:** Amino acid composition of the anchovy viscera protein hydrolysates analyzed by HPLC [24].

Amino Acids (Symbol)	Amino Acids (g/100 g)
Isoleucine (ILE)	3.26 ± 0.07
Leucine (LEU)	6.87 ± 0.07
Lysine (LYS)	10.94 ± 0.05
Methionine (MET)	2.56 ± 0.02
Phenylalanine (PHE)	6.83 ± 0.05
Threonine (THR)	2.42 ± 0.02
Valine (VAL)	4.19 ± 0.04
Arginine (ARG)	8.83 ± 0.05
Glycine (GLY)	10.87 ± 0.03
Proline (PRO)	5.86 ± 0.05
Tyrosine (TYR)	3.34 ± 0.02
Alanine (ALA)	12.06 ± 0.04
Glutamic Acid (GLU)	11.77 ± 0.09
Other	10.20
Total	100

## Data Availability

The data presented in this study are available in [Abbate, J.M.; Macrì, F.; Arfuso, F.; Iaria, C.; Capparucci, F.; Anfuso, C.; Ieni, A.; Cicero, L.; Briguglio, G.; Lanteri, G. 2021. Anti-Atherogenic Effect of 10% Supplementation of Anchovy (*Engraulis encrasicolus*) Waste Protein Hydrolysates in ApoE-Deficient Mice. *Nutrients*. https://doi.org/10.3390/nu13072137].

## Supplementary Materials

The following are available online at https://www.mdpi.com/article/10.3390/nu13072137/s1, Figure S1: Body weight trends in mice during 12-weeks experimental study.
