# Supplementary material for: Anti-Atherogenic Effect of 10% Supplementation of Anchovy (Engraulis encrasicolus) Waste Protein Hydrolysates in ApoE-Deficient Mice
Source: Nutrients. 2021 Jun 22;13(7):2137. doi: 10.3390/nu13072137 (PMC8308468; doi:10.3390/nu13072137)
Supplement: Supplementary file 1 [file nutrients-13-02137-s001.zip › nutrients-1258754-supplementary.pdf]

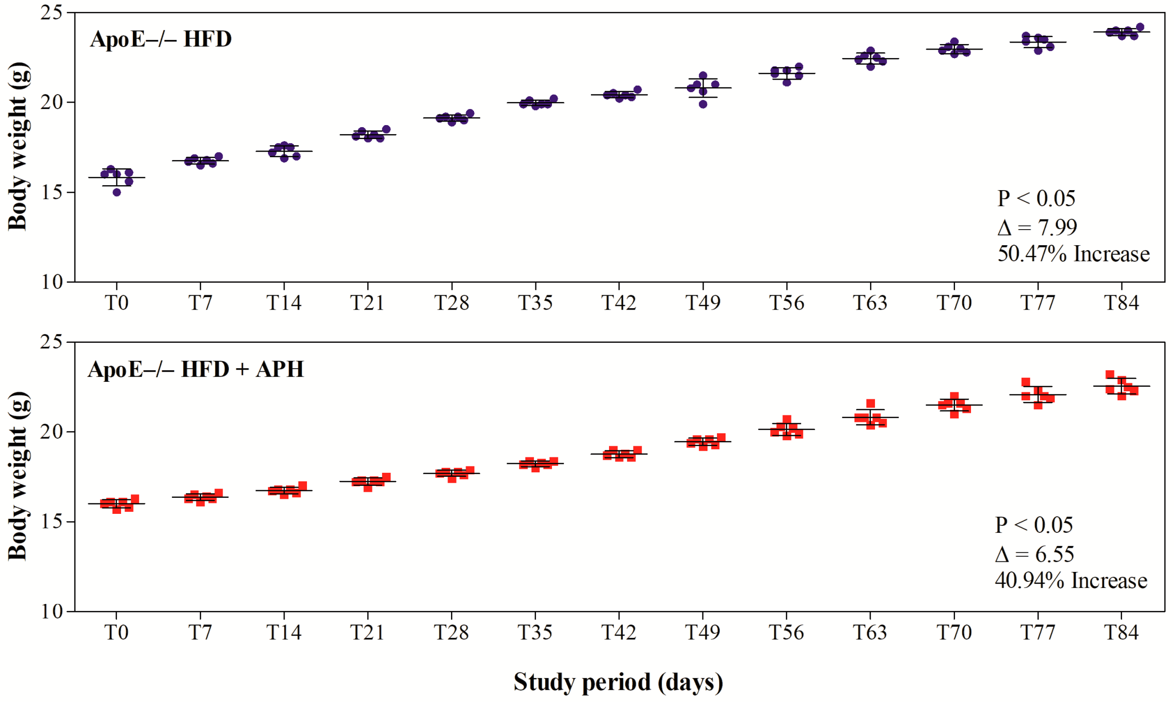

Figure S1. Body weight trends in mice during 12-weeks experimental study. An increasing trend in the body weight gain was observed in mice of both groups throughout the study (p <0.05), with a percentage increase of 50.47% recorded in ApoE$^{-/-}$ HFD and 40.94% in ApoE$^{-/-}$ HFD + APH (p < 0.001). Body weight gain was significantly lower in ApoE$^{-/-}$ HFD + APH compared to the control group, from study day 14, until the end of the study [25]
